# Trastuzumab resumption after extremely severe cardiotoxicity in metastatic breast cancer patient: a case report

**DOI:** 10.1186/s12885-017-3712-8

**Published:** 2017-11-07

**Authors:** Santino Minichillo, Ilaria Gallelli, Elena Barbieri, Marta Cubelli, Daniela Rubino, Sara Quercia, Massimo Dall’Olio, Claudio Rapezzi, Claudio Zamagni

**Affiliations:** 1 SSD Oncologia Medica Istituto “F.Addarii”, Sant’Orsola-Malpighi Hospital, University of Bologna, Via Massarenti, 9, 40138 Bologna, Italy; 2 Cardiovascular Department of the University of Bologna, Via Massarenti, 9, 40138 Bologna, Italy

**Keywords:** Trastuzumab, Cardiotoxicity, Monoclonal antibody, Breast cancer, Ejection fraction, Heart failure

## Abstract

**Background:**

Trastuzumab-related cardiotoxicity has been reported in patients receiving trastuzumab concurrently with other agents, especially with anthracyclines. Cardiac function damage is generally rare, precox and mild with trastuzumab alone.

**Case presentation:**

We report the case of a 49 year-old woman affected by metastatic breast cancer who developed trastuzumab-related cardiogenic shock due to pump failure (with LVEF of about 15%) after three months of treatment. After a long hospitalization in the cardiac intensive care unit and a proper treatment, LVEF increased to 50% and, due to a severe progression of disease, trastuzumab was resumed and continued for more than one year.

**Conclusion:**

This is a case of particularly severe cardiotoxicity related to trastuzumab treatment, which was recovered with pharmacological treatment and the temporary discontinuation of the treatment. Trastuzumab was safely resumed after clinical and echocardiographic parameters improvement.

## Background

Trastuzumab is a humanized monoclonal antibody that links the extracellular domain of the HER-2 protein (HER-2/neu or erbB2) overexpressed in about 20% to 25% of breast cancers. This antibody mediates signaling pathways leading to increased proliferation. It is approved for the treatment of early and metastatic breast cancer [1–3]. In HER-2 positive breast cancer, trastuzumab administered concurrently with chemotherapy has deeply modified the natural history of the disease by significantly improving response rates and survival in patients receiving chemotherapy and trastuzumab (CT + TR) compared to those receiving chemotherapy alone (CT) (median survival 25.1 months in CT + TR group versus 20.3 months in CT group, *P* 0.046) [3].

However, trastuzumab use has been found to be associated with an high incidence of cardiotoxicity which varies in the available literature from 0.6% to 4.5% reaching up to 34% when associated with anthracyclines [4].

The pathogenesis of trastuzumab-associated cardiac function decrease is still unknown and its mechanism of action is under investigation in small clinical studies. Several trials have described potential risk factors such as age, weight and high body mass index (BMI), history of coronary artery disease and hypertension, cumulative doxorubicin dose, HER2 expression level, previous treatment, radiation of the chest and negative hormonal receptor status [5]. However, among these, only age and concomitant doxorubicin therapy result to correlate with an increased risk of cardiotoxicity [3]. Moreover, despite the publication of clinical guidelines for the management of trastuzumab-induced cardiomyopathy, the choice to resume trastuzumab therapy after a decline in left ventricular ejection fraction remains a clinical decision based on expected risks and benefits.

Here we report the case of a metastatic breast cancer patient treated with trastuzumab associated to chemotherapy. She developed extremely severe congestive heart failure requiring complex specialized treatment. After full resolution of symptoms and left- ventricle ejection fraction (LVEF) recovery with appropriate therapy, she resumed, because of disease progression, trastuzumab treatment without any further cardiologic complications.

Few similar cases have been reported in the scientific literature but this case report is particularly interesting because the patient never received anthracyclines and, after resumption, trastuzumab was continued for about two years without LVEF alterations, resulting in complete remission of visceral neoplastic disease.

## Case presentation

In December 2000, a 49 year-old woman underwent left mastectomy for a stage IIA invasive ductal breast carcinoma with low proliferative activity (Ki 67 < 5%), negative hormone receptors and HER2 overexpressed (score 3+ at immunochemistry). In her medical history there were no cardiovascular comorbidities and she had no family history of cardiovascular disease.

From February to July 2001 she received an adjuvant chemotherapy with cyclophosphamide 600 mg/sqm, methotrexate 40 mg/sqm and 5-fluorouracyl 600 mg/sqm days 1,8. Subsequent follow-up was negative until September 2005 when a local left axillary relapse was resected. Histological and biological features of the relapse did not change. Surgical resection was followed, from January to February 2006, by radiation therapy on the left chest wall (5000 cGy with fractioned dose of 200 cGy/day). In November 2010, a PET-CT scan was performed to test for progressive increase in serum biomarkers. It showed multiple secondary localizations: lymph-nodal metastases (left axillary, mediastinic, iliac and lombo-aortic), liver metastases (third segment), and bone lesions (left seventh rib and left femur acetabulum). Liver biopsy confirmed hormone receptors negativity and HER2 overexpression (score 3+). The patient was absolutely asymptomatic (ECOG 0). A screening echocardiogram (January 2011) found no pathological findings and a normal left ventricular ejection fraction (LVEF 64%). At that point, first line chemotherapy with weekly paclitaxel (80 mg/sqm) associated with weekly trastuzumab (loading dose of 4 mg/kg followed by maintenance dose of 2 mg/Kg) was initiated and paclitaxel was withdrawn at the second administration because of hypersensitivity reaction and replaced with docetaxel (100 mg/sqm every three weeks). A supportive therapy with bisphophonates (zoledronic acid 4 mg i.v. every 28 days) was also administered for bone metastases. In March 2011, after three months of treatment (fourteen administrations of weekly trastuzumab), the patient referred asthenia, tachycardia, increasing dyspnea for mild efforts and palpitations. Within few days clinical conditions rapidly worsened and the patient was admitted to the emergency room for cardiogenic shock (heart rate 150 beats per minute, blood pressure 70/50 mmHg, severe oliguria, pulmonary congestion, NYHA 4, AHA D). An angio-CT scan excluded a pulmonary thromboembolism and the patient was admitted to a cardiac intensive care unit where an echocardiogram revealed a severe global biventricular dilatation and dysfunction (LVEF about 15%). Despite a maximal supportive therapy with inotropic agents and diuretics, shock persisted. Therefore an intraortic balloon pump was implanted with a very slow but progressive hemodynamic improvement and a resumption of diuresis. In absence of previous clinical experiences or data from the literature describing of similar serious clinical presentation using trastuzumab alone (without current or previous history of anthracyclines exposure), a myocardium biopsy was performed finding inflammatory areas of uncertain etiology but not compatible with a myocarditis. After approximately two months of hospitalization the patient was progressively weaned by inotropic agents and infusional diuretic therapy, and a heart failure pharmacological treatment was orally introduced (bisoprolol 2,5 mg, enalapril 2,5 mg, ivabradine 15 mg, canreonate 50 mg, furosemide 100 mg). After four months and with a slow pharmacological up-titration, we observed a progressive clinical improvement and an increase and stabilization of biventricular function (LVEF 45% on September 2011). In May 2012, a PET-CT scan showed lymph-nodal, liver and skeletal disease progression, at which point a second line chemotherapy with vinorelbine (25 mg/sqm, days 1,8) was initiated. In July 2012, after two cycles of chemotherapy, a further tumor assessment documented visceral disease progression. Therefore, with awareness of the high risk due to recent severe cardiogenic shock and after discussion with the patient and her family, we decided to resume trastuzumab therapy along with cardiac therapy. Weekly trastuzumab (2 mg/kg) was resumed with a clinical and echocardiographic cardiac monitoring. Soon thereafter, radiological evaluations (PET-CT scan and total body CT scan) showed a partial response of visceral disease. Trastuzumab and vinorelbine therapy was continued until June 2013 when, considering the positive response and the appearance of grade 2 neuropathy, vinorelbine was interrupted and trastuzumab was continued every 21 days (6 mg/Kg) until April 2014 with no further signs or symptoms of heart failure. External beam radiotherapy on left ileo-pubic branch (March 2012, 30 Gy) and on the second cervical vertebra (January 2013, 2000 cGy for 5 fractions) were performed for palliative purpose. Zoledronic acid every 28 days was continued during the whole period. Serial echocardiograms performed during trastuzumab treatment did not reveal LVEF drop maintaining in the range of 50–55%.

In January 2014 the patient presented diplopia, left eye squint, postural instability and leg weakness. A CT scan and MRI (February 2014) revealed the presence of four cerebral and cerebellar lesions (right cerebellar tonsil, left frontal and parietal lobe, quadrigeminal plate). The patient underwent stereotactic gamma-knife radiosurgery on February 2014 followed by whole brain irradiation for suspected leptomeningeal involvement (3000 cGy for 12 fractions). In April 2014 trastuzumab was interrupted and a new line of chemotherapy with capecitabine 3000 mg/day (1–14 every 21 days) associated with tyrosine kinase inhibitor (TKI) lapatinib 250 mg 4 tb/day was started. Subsequent radiological assessment (August 2015) documented a complete remission of lymph-nodal and liver neoplastic disease and radiologic stability of bone metastases.

**Fig. 1:**
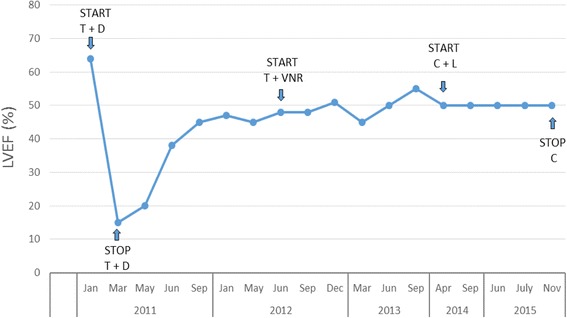
Left ventricular ejection fraction values in the treatment period. T: trastuzumab, D: docetaxel, VNR: vinorelbine, C: capecitabine, L: lapatinib

In November 2014 the patient was hospitalized for acute renal failure secondary to dehydration, fainting and recurrent vomiting. After discharge, she complained of severe asthenia, anorexia and weight loss. Consequently, chemotherapy with capecitabine was suspended and lapatinib was continued as monotherapy for about a year during which she never reported cough, dyspnea, chest pain and other symptoms suggestive of heart failure (NYHA II, AHA C). In November 2015, echocardiographic examination showed no relevant changes compared to the previous ones. We found an interesting pattern of LVEF trend along with trastuzumab treatment – discontinuation – and resumption (Fig. 1).

Over the following three months, the patient experienced a rapid deterioration of general clinical conditions, with progressive and definitive bedrest and altered conscience, suggesting a meningeal disease progression. In January 2016, the last ultrasound examination showed a progressive liver disease. The patient died on February 24, 2016 from neoplastic disease progression.

## Discussion

In this case report we described the case of a patient treated with trastuzumab who developed a severe congestive heart failure during treatment and who resumed trastuzumab therapy after proper cardiac management and left ventricular ejection fraction recovery.

Similarly to our case, Castells and colleagues reported the case of a breast cancer patient who developed a severe heart failure (LVEF 23%) after adjuvant treatment with doxorubicin and trastuzumab. Her cardiac function improved after positioning of a left ventricular axial pump. She fully recovered after 135 days of support therapy and the axial pump was successfully removed [6]. Conversely to our experience this patient did not need any further trastuzumab treatment.

The literature includes three cases similar to our experience. In the first report Hermann and colleagues described two breast cancer patients who had received trastuzumab monotherapy and were hospitalized for a rapidly worsening heart failure following hypertensive crisis, in one case due to the interruption of antihypertensive drugs and in the other case to an underlying sclerodermia. In both cases trastuzumab therapy was safely resumed after adequate treatment of the underlying causes of the heart failure [7].

Martin and colleagues reported on a patient with a left ventricular ejection fraction drop from 76% to 55% during adjuvant sequential anthracycline-containing chemotherapy followed by trastuzumab-based therapy; ejection fraction was restored after starting therapy with the ACE-inhibitor captopril, and trastuzumab was resumed without complications [8].

Unlike anthracycline-induced cardiotoxicity, trastuzumab cardiotoxicity has some peculiar features: 1) the risk does not seem to be dose related [9]; 2) there is no evidence that cardiac damage is associated with ultrastructural changes in myocytes and 3) it is fully reversible after treatment suspension [10]. Acute congestive heart failure due to anthracycline-induced cardiotoxicity is often serious and require a long-term hospitalization with a multidisciplinary approach. In contrast, trastuzumab-related cardiotoxicity is less severe and at least partially reversible in many cases [3].

Cardiotoxicity occurs more frequently in patients with preexisting hypertension and obesity, former or current smokers, with cardiologic comorbidities and with a family history of cardiovascular disease. Our patient had no cardiological comorbidities, she had not received anthracycline-based therapies although radiation therapy on the left chest wall was performed five years before. Tumor hormonal receptors, another risk factor, were also negative.

Typically, there is a rapid improvement in clinical conditions and ejection fraction after trastuzumab discontinuation and standard treatment allowing resumption of trastuzumab, as we did for our patient.

In the present report our patient developed cardiogenic shock due to a severe biventricular dysfunction probably caused by trastuzumab four months after the beginning of the treatment. One year later, after a full recovery from heart failure, because of disease progression, and with consideration of the clinical benefits from anti-HER2 treatment and the absence of absolute cardiologic contraindications (LVEF was significantly improved with proper treatment), therapy with full dose trastuzumab was resumed along with a close monitoring program of left ventricular function for about two years without any further cardiovascular complications. In summary, our patient completed a line of chemotherapy with trastuzumab. Subsequently, we performed 12 cycles with trastuzumab (6 mg/Kg) alone which led to a complete remission of visceral disease.

The question of how best to prevent trastuzumab-related cardiotoxicity continues to be debated. Since the exposure to both trastuzumab and an anthracycline leads to the greatest risk, guidelines recommend avoiding concurrent exposure to these two agents. Another strategy could be to identify patients who are at greater risk of cardiac complications from trastuzumab therapy by using a radiolabeled trastuzumab (111In-DTPA-trastuzumab) and by evaluating scintigraphic myocardial uptake [11]. Other than a study by Cardinale et al., there is little data from the literature on the utility of cardiac biomarkers for trastuzumab, such as TnI [12]. Increase of TnI was found exclusively in patients pre-treated with anthracyclines and in seven patients was reported prior to trastuzumab exposure, suggesting possible additional effects on a previous anthracycline-induced myocyte injury.

Although this is only a single case-report we believe it to be valuable because the cardiac function of our patient recovered, despite the severity of heart failure without a previous exposure to anthracyclines, following adequate cardiac management, allowing her to benefit from prolonged trastuzumab treatment followed by additional anti-HER2 agent lapatinib treatment.

## Conclusions

Trastuzumab can lead to a severe cardiac toxicity, including in patients never previously exposed to anthracyclines and irrespective of other risk factors. It is usually reversible and, when recognized early, responds to the discontinuation of the antibody and to standard heart failure treatment, providing the opportunity for resumption of therapy if necessary.

## Abbreviations

ACE: Angiotensin converting enzyme
AHA: American Heart Asssociation
BNP: B-type natriuretic peptide
CGy: Centigrays
CK: Creatinchinase
CK-MB: Creatinchinase MB
CT: Computed tomography
DTPA: Diethylene triamine pentaacetic acid
Gy: Grays
LVEF: Left ventricular ejection fraction
MRI: Magnetic resonance imaging
MUGA: Multi gated acquisition scan
NYHA: New York Heart Association
PET: Positron emission tomoscintigraphy
TnI: I troponin
TnT: T troponin
